# Incidence and predictors of death from COVID-19 among patients admitted to treatment center of Wollega University Referral Hospital, Western Ethiopia: A retrospective cohort study

**DOI:** 10.1371/journal.pone.0267827

**Published:** 2022-07-27

**Authors:** Tadesse Tolossa, Bizuneh Wakuma, Diriba Ayala, Dejene Seyoum, Getahun Fetensa, Ayantu Getahun, Diriba Mulisa, Emiru Merdassa Atomssa, Reta Tsegaye, Tesfaye Shibiru, Ebisa Turi, Lami Bayisa, Ginenus Fekadu, Balay Bekele, Ilili Feyisa

**Affiliations:** 1 Department of Public Health, Institute of Health Sciences, Wollega University, Nekemte, Ethiopia; 2 Department of Nursing, Institute of Health Sciences, Wollega University, Nekemte, Ethiopia; 3 Department of Midwifery, Institute of Health Sciences, Wollega University, Nekemte, Ethiopia; 4 Department of Health Behavior and Society, Institute of Health, Jimma University, Jimma, Ethiopia; 5 Department of Pediatrics, School of Medicine, Wollega University, Nekemte, Ethiopia; 6 Department of Pharmacy, Institute of Health Sciences, Wollega University, Nekemte Ethiopia; 7 Faculty of Medicine, School of Pharmacy, The Chinese University of Hong Kong, Shatin, N.T, Hong Kong; 8 Wollega University Referral Hospital, Institute of Health Sciences, Wollega University, Nekemte, Ethiopia; Ohio State University, UNITED STATES

## Abstract

**Introduction:**

Currently, COVID-19 contributes to mortality and morbidity in developed as well as in developing countries since December 2019. However, there is scarcity of evidence regarding the incidence and predictors of death among patients admitted with COVID-19 in developing country including Ethiopia, where the numbers of deaths are under-reported. Hence, this study aimed to assess the incidence and predictors of death among patients admitted with COVID-19 in Wollega University Referral Hospital (WURH), western Ethiopia.

**Methods:**

An institution based retrospective cohort study design was conducted among 318 patients admitted with COVID-19 in WURH treatment center. Patients who were tested positive for COVID-19 by using rRT-PCR test and admitted with the diagnosis of severe COVID-19 cases from September 30, 2020 to June 10, 2021 were a source population. Epidata version 3.2 was used for data entry, and STATA version 14 for analysis. A Cox proportional hazard regression analysis was used to determine factors associated with mortality from COVID-19. Multivariable Cox regression model with 95% CI and Adjusted Hazard Ratio (AHR) was used to identify a significant predictor of mortality from COVID-19 at p-value < 0.05.

**Results:**

A total of 318 patients were included in final analysis with mean age of 44 (SD±16.7) years and about two third (67.9%) were males. More than half (55.7%) of patients had no comorbidity on admission. The majority, 259 (81.45%) of patients recovered from COVID-19 and 267 (84%) of patients were censored at the end of follow up. The incidence rate of mortality was 14.1 per/1000 (95%CI: 10.7, 18.5) person days observation. Age ≥ 59 years (AHR: 5.76, 95%CI: 2.58, 12.84), low oxygen saturation (AHR: 2.34, 95% CI: (2.34, 4.17), and delayed presentation (AHR: 5.60, 95%CI: 2.97, 10.56) were independent predictors of mortality among COVID-19 patients.

**Conclusion:**

The mortality rate of COVID-19 pandemic was high in the study area, and most of death was happened during the first 10 days. Being old age, low oxygen saturation and delayed presentation were factors which predict mortality due to COVID-19. Hence, strengthening the health care delivery system to satisfy the need of the patients should get due attention to reduce the incidence of mortality from COVID-19 cases.

## Introduction

The epidemiological dynamics of Covid-19 has changed dramatically over the course of months. Globally 3.97 million people died out due to Covid-19. The number of infected people in Africa was nearly 5 million, and the pandemic is trending upwards still [1] In many countries, the number of cases and number of deaths are highly increasing, as a result of second wave or may be the third wave, low access to vaccine, and prolonged pandemic effects of the disease [2].

Evidence from Turkish population indicated that hospital mortality rate due to COVID-19 was 4.5% [3]. In additional result from Mexico revealed that incidence of death due to COVID-19 ranges from 7 to 15,929 per million with varying rate of mortality between under-20 age groups and in the older age groups [4], and the standardized mortality rate among population infected with COVID-19 in prison was 199.6 death per 100000 population in US [5].

“Death due to COVID-19 is a death resulting from a clinically compatible illness, in probable or confirmed COVID-19 case, unless there is a clear alternative cause of death that cannot be related to COVID disease” [6]. A recent World Health Organization (WHO) report indicated that death from COVID-19 in Africa had increased by 40% at the second wave, pushing Africa’s death total towards 100,000as the continent \battles’ new and more contagious variants [7].

A nation-wide systematic review and meta-analysis conducted in mid-2020 showed that, the pooled prevalence of in hospital mortality in patients with coronavirus disease in Ethiopia was 5% [8]. In Ethiopia the number of death during the first quarter of 2020 was 3747 in a single treatment center [9].

Variables like male sex, severe pneumonia, multi-organ dysfunction, sepsis, malignancy, interstitial lung diseases were identified as risk factor for Covid-19 [3]. Furthermore mortality due to COVID-19 was high and associated with older age, ICU admission, heart disease and liver disease [10]. The coexistence of DM with COVID-19 is the major contributing factor for the increased severity and mortality [11–13]. Additionally obesity, kidney disease, an average time in days of late consultation, dementia, and peripheral oxygen saturation were strong leading factors for death from COVID-19 [14,15].

As the second wave beating Africa cases surged far beyond the peak experienced in the first wave, health facilities have become overwhelmed. Preliminary WHO report shows that 66% reported inadequate critical care capacity, 24% reported burnout among health workers and some countries reported that oxygen production remains insufficient [7]. The COVID-19 induced mortality rate varies among countries and affected by a number of risks factors, and therefore, this study was designed to assess survival status and its predictors of admitted COVID-19 patients in treatment center of WURH, western Ethiopia.

## Methods

### Study setting, period and design

The study was conducted at Wollega University referral (WURH) hospital COVID-19 treatment center which is found in Nekemte town. Nekemte is the capital city of Eastern Wollega Zone and it is found 330 km away from Addis Ababa, the capital city of the country. WURH treatment center is one of the treatment center found in Western part of the country established under Federal Ministry of Health, since the first case of COVID-19 was reported in Ethiopia. In collaboration with other partner, WURH treatment center have actively participated in combating the spread of COVID-19 pandemic through awareness creation, laboratory diagnosis, and admission of confirmed cases. The treatment center has been initiated laboratory testing for COVID-19 cases, established isolation center and quarantine center for suspected cases, and serving as treatment center for confirmed cases. Patients have been managed in the separate ward established for this purpose and ICU ward for critical cases. All health professionals with different backgrounds such as Nurses, Physician, Pharmacy, Medical Laboratory Technologist, Biomedical and Radiologists have been participated in treating and giving support to patients. Equipment like patient monitoring devices, pulse Oximetry, oxygen cylinder, mechanical ventilator, thermometer, oxygen concentrator, pack machine, flow meter, and stretcher are found in the center. Since its establishment, a total of 732 COVID-19 cases were admitted to WURH treatment center. From end of March 2020 (the day when the first case admitted to treatment center) to end of September 2020, all COVID-19 cases were admitted to the treatment center regardless of the severity of the disease. Since end of September 2020, the treatment center started to admit only severe cases of COVID-19. Asymptomatic cases of COVID-19 have been discharged and advised for self-isolation at their home.

The study period was from September 30, 2020, to June 10, 2021. The data were retrieved between June 10, 2021 and June 25, 2021. A facility based retrospective cohort study design was employed.

### Study population, eligibility criteria and sampling techniques

Patients who were tested positive for COVID-19 by using rRT-PCR test and admitted with the diagnosis COVID-19 cases during study period where study population. Patients with incomplete outcome variable and important baseline information were excluded from the analysis. All COVID-19 patients admitted to the treatment centers during study period (September 30, 2020, to June 10, 2021) and fulfill inclusion criteria were included in the analysis. With convenient sampling technique, all eligible patients were included in the analysis.

#### Study variables and outcome measures

The dependent variable of this study was incidence of mortality from COVID-19. Incidence of mortality from COVID-19 was those patients who were died as a result of COVID-19 while they are on treatment during the follow-up period. The outcome variable (incidence of mortality) was death of patients from severe COVID-19 while they were in the treatment center, and death recorded on card was ascertained by physician on duty. The survival time was estimated in days, and it was the time when the patient was diagnosed positive for COVID 19 by using rRT-PCR test to the patient was developed the outcome (event/censored). Censored were those patients who were not developed an event or not dead from COVID-19 (recovery, refused treatment, on treatment when the study was completed).

Socio demographic variables such as age, sex, residence; clinical related variables like presence of co-morbidity, types of comorbidity, types of clinical manifestation on admission, duration of the clinical manifestation on arrival to the hospital; laboratory variables such as random blood sugar (RBS), haemoglobin, oxygen saturation; types of medications prescribed for the patients and types of complication happened while the patients was on follow-up were selected as an independent variables for this study.

Comorbidity was co-existence of chronic non-communicable diseases such as coronary artery disease, chronic obstructive pulmonary disease (COPD), and diabetes or disabilities [16,17]. Oxygen saturation was categorized as normal oxygen levels in a pulse oximeter which is range from 94% to 100%, and blood oxygen levels below 94% are considered low (hypoxemia).

Severity of the disease was categorized into Asymptomatic for individual who have no symptoms that are consistent with COVID-19. Mild Illness was when an the existence of any of the various signs and symptoms of COVID-19 (e.g., fever, cough, sore throat, malaise, headache, muscle pain, nausea, vomiting, diarrhea, loss of taste and smell) but who do not have shortness of breath, dyspnea, or abnormal chest imaging. Moderate Illness was defined as an evidence of lower respiratory disease during clinical assessment or imaging and who have oxygen saturation (SpO_2_) ≥94% on room air at sea level. Severe Illness was when an individual’s manifests oxygen saturation (SpO_2_) <94% on room air at sea level, a respiratory rate >30 breaths/min, or lung infiltrates >50%. Critical Illness was reported when an individual’s shows an evidence of respiratory failure, septic shock, and/or multiple organ dysfunctions [18].

#### Data collection tools and procedure

Data were collected from medical cards of COVID-19 patients. The checklist for data extraction tool consists of socio demographic related variables, disease related variables, clinical and laboratory related variables. Health professionals who have been working in the treatment center was retrieved the data. Prior to data collection period, one day training was given for the data collectors. During data collection time, the outcome was confirmed by reviewing the chart which was recorded by physician. The investigators also supervised overall data collection.

### Data management and analysis

Epi-data version 3.2 was used for data entry, and then the data was exported to STATA version 14 for further analysis. Before analysis, data was cleaned, edited by using simple frequencies and cross tabulation. Days were used as time scale to calculate median time to death. Incidence of mortality was estimated by dividing total number of new death by total person day observation (PDO). Person days observation (PDO) was calculated as date of outcome (event or censored) happened subtracted from date of COVID-19 diagnosis by using rRT-PCR test. Descriptive non-parametric survival analysis such as Kaplan Meier failure curve was used for the estimation failure probability. Log rank test was used to test any difference in failure probability in categorical covariates.

A cox proportional hazards regression model was used to determine factors associated with incidence of mortality. Factors associated with incidence of mortality at p-value < 0.25 in bivariable cox regression were selected for multivariable cox regression analysis. Adjusted Hazard Ratios (AHR) with 95% confidence intervals was computed and statistical significance was declared when it is significant at 5% level (p-value < 0.05). A proportional hazard assumption was checked by using by log-log plot and global test. Overall model adequacy of proportional hazard model was assessed by using coxsnell residual graph.

### Ethical approval and informed consent

Ethical clearance was obtained from Wollega University Institute of Health Sciences research review committee. Formal letter of cooperation was written to WURH treatment center and permission was obtained from the hospital administration. Informed consent was obtained from all the participants or responsible third-party caregivers. Personal identifiers were not used on data collection checklist. To ensure confidentiality, name and other identifiers of participants and health care professionals were not recorded on the data collection tools. All methods were performed in accordance with the relevant guidelines and regulations.

## Results

From September 30, 2020 to June 10, 2021, a total of 417 patients with severe COVID-19 were admitted to WURH treatment center. Of these, 99 patient cards were excluded from analysis due to unregistered outcome (event, censored, date of admission, date discharge and other baseline data incomplete). Finally, 318 patient cards with complete data were included in final analysis.

The mean age of participants was 44 (SD±16.6) years with minimum and maximum age of 11 and 92 years, respectively. The majority 177 (55.7%) of the participants age were between 25–58 years. More than half (58.5%) of patients were resides in urban areas, and more than two third (67.9%) of participants were males (Table 1).

**Table 1:** Socio-demographic characteristics of COVID-19 cases admitted to Wollega University Referral Hospital, Western Ethiopia.

Variables	Category	Outcome		Total No (%) (n = 318)
		Dead (Event) No (%)	Survived (Censored) No (%)	
Age	≤24 years 25–58 years ≥58 years	6 (9.9) 8 (9.5) 37 (23.9)	42 (90.1) 169 (90.5) 56 (76.1)	48 (15.1) 177 (55.7) 93 (29.2)
Sex	Male Female	35 (16.2) 16 (15.7)	181(83.8) 86 (84.3)	216 (67.9) 102 (32.0)
Residence	Urban Rural	32 (17.2) 19 (14.4)	154 (82.8) 113 (85.6)	186 (58.5) 132 (41.5)

### Clinical characteristics of COVID-19 admitted cases

**Fig 1:**
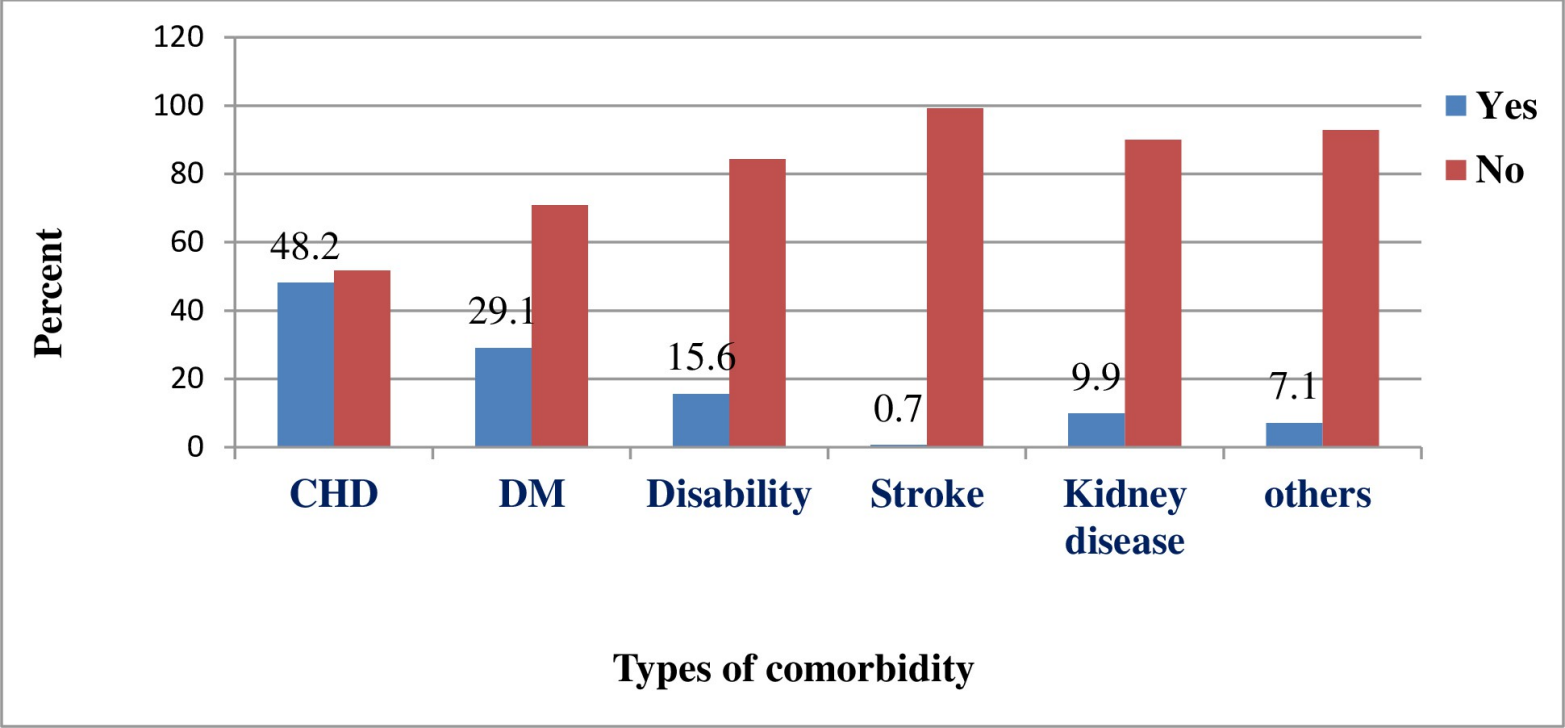
Types of comorbidity among patients admitted with severe COVID-19 cases in WURH treatment center, Western Ethiopia, 2021.

More than half (55.7%) of patients had no comorbidity on admission. The main comorbidity identified during admission was coronary artery disease (48.2%) followed by diabetes mellitus (29.1%) (Fig 1). Fever was manifested on around three fourth (74.2%) of patients, while sore throat was not complained by majority (92.1%). According to this study, 16 (5%) of participants have experienced psychological distress and more than half (55.3%) of patients were subcritical on admission. Two hundred sixty (67.9%) of cases had experienced clinical manifestation of 1–7 days on arrival to the hospital (Table 2).

**Table 2:** Clinical characteristics of COVID-19 cases admitted to Wollega University Referral Hospital treatment center, Western Ethiopia.

Clinical characteristics	Category	Outcome		Total No (%)
		Dead (Event) No (%)	Survived (Censored)No (%)	
Comorbidity on admission	No Yes	13 (7.3) 38 (27.0)	164 (92.7) 103 (73.0)	177 (55.7) 141 (44.3)
Fever on admission	No Yes	6 (7.3) 45 (19.1)	76 (92.7) 191 (80.9)	82 (25.8) 236 (74.2)
Headache on admission	No Yes	14 (13.3) 37 (17.4)	91 (86.7) 176 (82.6)	105 (33.0) 213 (67.0)
Chest pain on admission	No Yes	10 (6.6) 41 (24.7)	142 (93.4) 125 (75.3)	152 (47.8) 166 (52.2)
Loss of appetite on admission	No Yes	10 (7.1) 41 (23.0)	130 (92.9) 137 (77.0)	140 (44.0) 178 (56.0)
Sore throat	No Yes	46 (15.7) 5 (20.0)	247 (84.3) 20 (80.0)	293 (92.1) 25 (7.9)
Cough on admission	No Yes	0 (0.0) 51 (17.1)	20 (100.0) 247 (82.9)	20 (6.3) 298 (93.7)
Shortness of breath	No Yes	0 (0.0) 51 (19.9)	62 (100.0) 205 (80.1)	62 (19.5) 252 (80.5)
Fatigue on admission	No Yes	14 (10.9) 37 (19.6)	115 (89.1) 152 (80.4)	129 (40.6) 189 (59.4)
Status of patient on admission	Stable Subcritical Critical	6 (10.3) 21 (11.9) 24 (28.6)	52 (89.7) 155 (88.1) 60 (71.4)	58 (18.2) 176 (55.3) 84 (26.4)
Organ failure	No Yes	41 (13.5) 10 (71.4)	263 (86.5) 4 (28.5)	304 (95.6) 14 (4.4)
Duration of C/M on arrival	1–7 days >7 days	22 (10.2) 29 (28.4)	194 (89.8) 73 (71.6)	216 (67.9) 102 (32.1)

C/M: Clinical Manifestation.

### Medication prescribed and laboratory investigation

Patients admitted with COVID-19 cases have been taking medication ranging from intra-nasal oxygen to Vancomycin. Accordingly, 84.6%, 17.6%, 45.0%, 64.8%, 93.4% have been taking anti-pain/analgesics, Heparin, Vancomycin, Azithromycin, and intra nasal oxygen, respectively. Majority, (93.4%) patients have been received intra nasal oxygen during their hospital stay. More than three fourth (79.9%) of admitted COVID-19 cases had RBS level greater than 200 mg/dl (Table 3).

**Table 3:** Types of medication prescribed for patients admitted with COVID-19 cases in WURH, Western Ethiopia.

Variables	Category	Outcome		Total No (%)
		Dead (Event) No (%)	Survived (Censored)No (%)	
Anti-pain/analgesics	No Yes	3 (6.1) 48 (17.8)	46 (93.9) 221 (82.2)	49 (15.4) 269 (84.6)
Dexamethasone	No Yes	8 (5.9) 43 (23.6)	128 (94.1) 139 (76.4)	136 (42.8) 182 (57.2)
Heparin	No Yes	31 (11.8) 20 (35.7)	231 (88.2) 36 (64.3)	262 (82.4) 56 (17.6)
Ceftazidine	No Yes	22 (10.2) 29 (28.4)	194 (89.8) 73 (71.6)	216 (67.9) 102 (32.1)
Cefexime	No Yes	46 (16.2) 5 (14.7)	238 (83.2) 29 (85.3)	284 (89.3) 34 (10.7)
Vancomycin	No Yes	18 (10.3) 33 (23.1)	157 (89.7) 110 (76.9)	175 (55.0) 143 (45.0)
Ceftriaxone	No Yes	32 (21.6) 19 (11.2)	116 (78.4) 151 (88.8)	148 (46.5) 170 (53.5)
Azithromycin	No Yes	25 (77.7) 26 (12.6)	87 (22.3) 180 (87.4)	112 (35.2) 206 (64.8)
Lasix	No Yes	23 (9.8) 28 (33.3)	211 (90.2) 56 (66.7)	234 (73.6) 84 (26.4)
Intra nasal oxygen	No Yes	1 (4.8) 50 (16.8)	20 (95.2) 247 (83.8)	21 (6.6) 297 (93.4)
Oxygen saturation	< 94% ≥ 94%	18 (75.0) 33 (11.2)	6 (25.0) 261 (88.8)	24 (7.6) 294 (92.4)
RBS level	≤200 mg/dl >200 mg/dl	29 (11.4) 22 (34.4)	225 (86.6) 42 (65.6)	64 (20.1) 254 (79.9)

### Encountered complications and diagnosed comorbidity state during hospital stay

Bedsore was the least (1.9%) complication registered during hospital stay while, respiratory distress (23.6%) was the major complication that was experienced by patients. Diabetes mellitus was diagnosed in 20 (6.3%) of patients, and loss of consciousness was seen on 12.3% of cases (Table 4).

**Table 4:** Types of complications developed on patients admitted with severe COVID-19 cases in WURH treatment center, Western Ethiopia.

Variables	Category	Outcome		Total No (%)
		Dead (Event) No (%)	Survived (Censored)No (%)	
Acute respiratory distress	No Yes	15 (6.2) 36 (48.0)	228 (93.8) 39 (52.0)	243 (76.4) 75 (23.6)
Deep vein thrombosis	No Yes	44 (14.4) 7 (58.3)	262 (85.6) 5 (41.7)	306 (96.2) 12 (3.8)
Acute kidney failure	No Yes	45 (14.6) 6 (66.7)	264 (85.4) 3 (33.3)	309 (97.2) 9 (2.8)
Diabetes mellitus (DM)	No Yes	45 (15.1) 6 (30.0)	253 (84.9) 14 (70.0)	298 (93.7) 20 (6.3)
Anemia	No Yes	44 (14.4) 7 (53.8)	261 (85.6) 6 (46.2)	305 (95.9) 13 (4.1)
Bed sore	No Yes	50 (16.0) 1 (16.7)	262 (84.0) 5 (83.3)	312 (98.1) 6 (1.9)
Electrolyte imbalance	No Yes	46 (15.3) 5 (27.8)	254 (84.7) 13 (72.2)	300 (94.3) 18 (5.7)
Psychological disorder	No Yes	46 (15.2) 5 (31.2)	256 (84.8) 11 (68.8)	302 (95.0) 16 (5.0)
Loss of consciousness	No Yes	26 (9.3) 25 (64.1)	253 (90.7) 14 (35.9)	279 (87.7) 39 (12.3)

### Survival status patients admitted with COVID-19 in WURH

A total of 318 patients were followed for a median follow up time of 6 days with Interquartile rage of 6–15 days. The majority, 259 (81.45%) of patients were recovered from COVID-19. A total of 267 (84%) of participants were censored at the end of follow up.

### Incidence rate of death and median time to death

**Fig 2:**
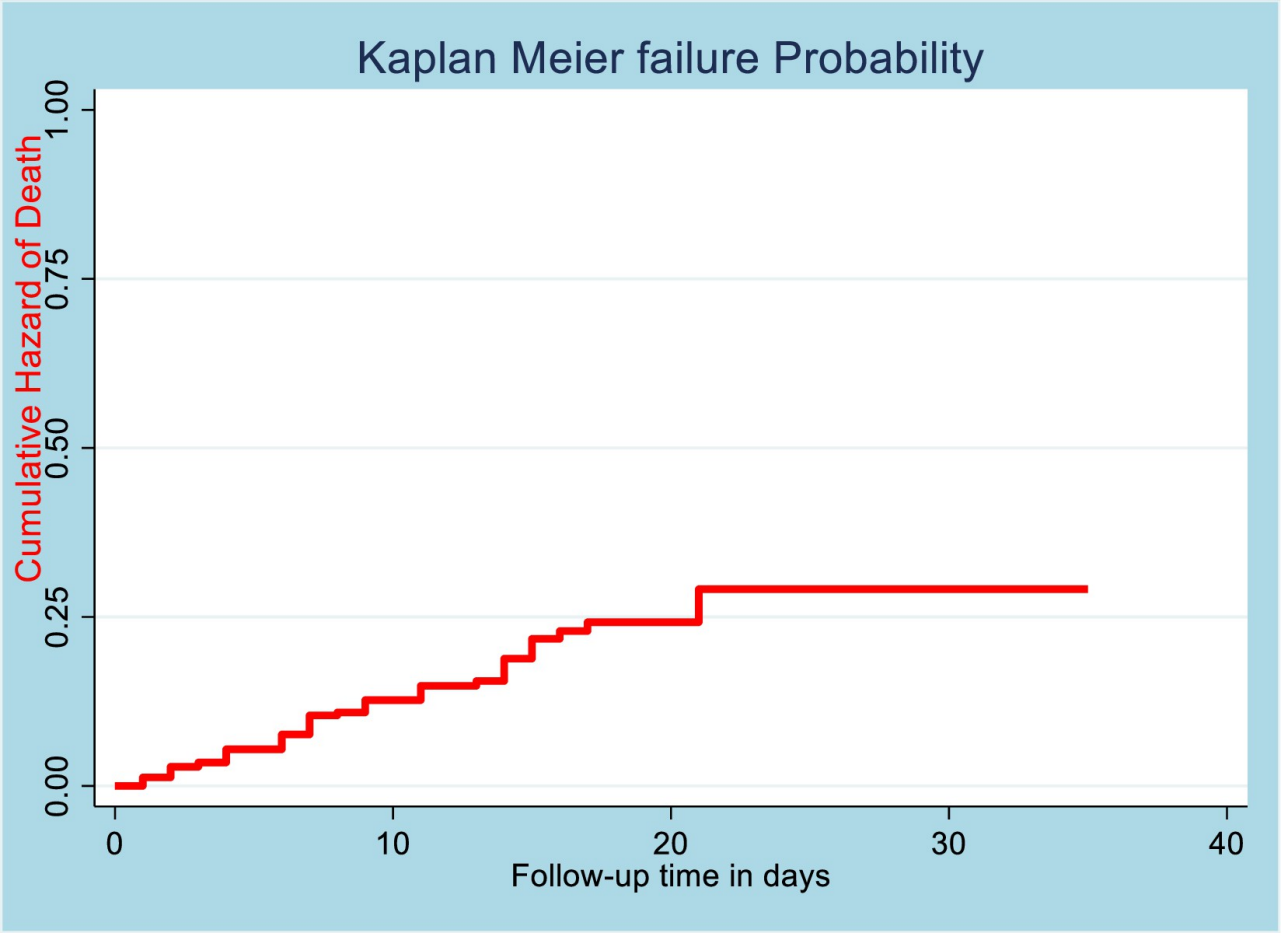
Overall Kaplan Meier failure probability curve.

The patients had 1–35 days follow up times giving total 3618 person days observations of death free periods. Overall, 51 (16%) patients were dead from COVID-19 and recorded as an event of the study. The incidence rate of mortality was 14.1 per/1000 (95%CI: 10.7, 18.5) person days. The median time to death from COVID-19 was 7 days (95% CI: 7, 9) days. The overall incidence rate of death was described by Kaplan Meier hazard curve, and it shows most of the death was happened during the first 20 days, and the rate of death was almost similar after 20 days of follow up (*Fig 2*).

### Bivariable and multivariable cox regression analysis

Residence, age of the patients, sex, level of oxygen saturation, duration of clinical manifestation before admission, and comorbidity on admission were found as a candidate for multivariable cox regression model. Finally, three variables (age, oxygen saturation, and duration clinical manifestation) were found to be statistically significant association mortality during multivariable cox regression analysis at 95% CI.

Age of patients showed statistically significant association with death, in which the risk death due to COVID-19 was about 5.76 times higher among patients aged older than 58 years when compared to patients aged 25–58 years (AHR:5.76, 95%CI: 2.58, 12.84). The hazard of death among patients who had low oxygen saturation (<94%) was 5.60 times higher than patients with normal oxygen saturation (AHR: 5.60, 95%CI: 2.97, 10.56). The hazard of death among patients presented to health institution after 7 days of clinical manifestation was 2.34 times higher when compared to individuals presented to health institution within 7 days of clinical manifestation (AHR:2.34, 95% CI: (2.34, 4.17) (Table 5).

**Table 5:** Multivariable cox regression analysis of predictors of mortality rate among patients admitted with severe COVID-19 in WURH treatment center.

Variables	Category	Outcome		CHR	AHR	P-value
		Dead (Event) No	Survived (Censored) No			
Sex	Female Male	16 35	86 181	Ref 1.01 (0.55, 1.82)	Ref 0.72 (0.39, 1.34)	0.306
Age	≤24 years 25–58 years ≥59 years	6 8 37	42 169 56	3.02 (1.05, 8.74) Ref 9.10 (4.23, 19.55)	3.69 (0.95, 10.85) Ref 5.76 (2.58, 12.84)	0.061 <0.001*
Residence	Urban Rural	32 19	154 113	Ref 0.86 (0.48, 1.51)	Ref 1.01 (0.56, 1.83)	0.220
Presence of comorbidity	No Yes	13 38	164 103	Ref 3.61 (1.92, 6.79)	Ref 1.99 (0.96, 3.94)	0.055
Oxygen saturation	≥ 94% < 94%	33 18	261 6	Ref 8.64 (4.86, 15.37)	Ref 5.60 (2.97, 10.56)	<0.001*
Duration of clinical manifestation	≤ 7 days >7 days	22 29	194 73	Ref 2.94 (1.68, 5.12)	Ref 2.34 (1.32, 4.17)	0.004*

AHR: Adjusted Hazard Ratio; CHR: Crude Hazard Ratio; *statistically significant at p ≤ 0.05, RBS-Blood Blood Sugar.

## Discussion

Despite the efforts that have been made to contain the spread of the virus and its consequences, COVID-19 continue to be a global public health challenge. Globally, the virus killed more than 3.9 million peoples by July 3, 2021 [19]. This study was conducted to assess the incidence rate of mortality and its predictors in Ethiopia where data related to mortality due to death was scarce.

The present study has shown that the median time to death from severe form of COVID-19 was 7 days. This was shorter period compared to the study done in Wuhan China which reported 12 days for the median time to death [20]. This discrepancy might be due to the relative difference in health care delivery system among the populations. The incidence rate of mortality was 14.1 per 1000 person-days’ observation and 16% of the patients were deceased. This was comparable to the study done in tertiary teaching hospital of Wuhan in which mortality rate ranges from (15–16.1%) [21,22].

Moreover, it is also comparable with the Global review and meta-analysis (15%) and another review and meta-analysis done in Asia, America and Europe (18.88%) [23]. However, it was lower than the study report from Spain (28%) [24], Taiwan(31.3%) [25], Seventh hospital of Wuhan city (23%) [26], New York (24%) [27], Wuhan Jinyintan Hospital in China (41.79) [28]. This could be due to the larger proportions of advanced age patients and comorbid conditions among COVID -19 patients in the previous studies. However, it was higher than the study done in Mainland China (3.1%) [29], Zhongnan Hospital of Wuhan University (4.3%) [30], Stony Brook University Hospital of New York City(5.45%) [31]. This might be due to the better quality of services delivered at intensive care unit of China and New York City than in the current study.

Regarding the predictors of mortality among COVID-19 patients, age, low oxygen saturation, and delayed presentation were independently predicted the hazard of death of COVID-19 cases. Accordingly, this study revealed that the hazard of death was fivefold higher among patients older than 59 years of age compared to those aged between 25–58 year which was supported by several previous studies [23,24,27,28,32,33]. This might be because degeneration of physiologic functions, pre-existing age related low immunity and increased risk of comorbidities with increased age, which further complicates the treatment outcome of COVID-19 patients [34]. Besides, the present study had also shown that the hazard of death was 5.7 fold increased among patients with low oxygen saturation compared to their counterparts. Existing pieces of evidence also supported this finding [24,33]. Low oxygen saturation is the manifestation of acute respiratory distress which needs supplementary oxygen. In our context, intensive care unit was not well established so that the unit cannot adequately respond to oxygen need of the patients and as a result patients may die. Moreover, patients with low oxygen saturation may need mechanical ventilation to sustain breathing which is very limited in resource limited settings like Ethiopia and eventually leading to death.

This study has also found delayed presentation after the onset of the symptoms as predictor of mortality from COVID-19 cases that the hazard of death was more than twofold higher among patients who presented after 7 days of the onset of clinical symptoms compared to those who sought medical advice before 7 days of the onset of clinical symptoms. This might be due to the fact that delay in seeking medical advice may complicate the disease condition and lead to poor treatment outcome like death.

### Limitation of the study

Current study used secondary data in absence of base-line data in which we forced to exclude data with un recorded outcome. Which in turn can reduce adequacy of sample. Furthermore, the factors that are stated in this study are limited to the factors those were on record only. Therefore factors contributing factors for incidence of covid-mortality were not limited to the factors those were stated within this study. Additionally, absence of standardized ICU care within the study area was factors that can affect the outcome of the study. study.

## Conclusion

In general, the incidence rate of mortality among COVID-19 patients was high in the study area. The median time to death was too short implying most patients used to seek medical advice after the disease complicates. The study also found greater than 58 years of age, low oxygen saturation, and delayed presentation as independent predictors of mortality among COVID-19 patients. Therefore, elders and patients with comorbid conditions have to get special attention during COVID-19 case management to enhance good treatment outcome. Furthermore, strengthening the health care delivery system to satisfy the need of the patients should get due attention to reduce the incidence of mortality from COVID-19 cases.

## Supporting information

S1 Dataset (DTA)
